# Comparative Transcriptomic and Proteomic Analyses Identify Byssogenesis-Associated Genes in the Mediterranean Mussel *Mytilus galloprovincialis* Lamarck, 1819

**DOI:** 10.3390/ijms262110511

**Published:** 2025-10-29

**Authors:** Xiuwei Zhen, Yiwen Chen, Wei Zhang, Yongren Li, Li Li, Haigang Qi, Shoudu Zhang

**Affiliations:** 1 Marine Science Research Institute of Shandong Province (National Oceanographic Center, Qingdao), Qingdao 266104, China; zhenxiuwei@163.com (X.Z.); ceng_an95@163.com (Y.C.); zhangwei02@shandong.cn (W.Z.); 2 College of Fisheries, Tianjin Agricultural University, Tianjin 300384, China; lyr1018@163.com; 3 Shandong Key Laboratory of Intelligent Marine Ranch (Under Preparation), Qingdao 266104, China; 4 Institute of Oceanology, Chinese Academy of Sciences, Qingdao 266071, China; lili@qdio.ac.cn

**Keywords:** *Mytilus galloprovincialis*, byssogenesis, transcriptomics, proteomics, gene

## Abstract

Mussels’ byssus and their adhesion ability play a crucial role in their attachment and artificial cultivation of mussels. In this study, transcriptomic and proteomic analyses were performed to identify byssogenesis-associated genes in the Mediterranean mussel *Mytilus galloprovincialis* Lamarck, 1819, seeking to advance our knowledge of the molecular basis of byssal secretion in mussels. Transcriptomic analysis identified 1742 and 1498 differentially expressed genes in the foot tissue of *M. galloprovincialis* at 9 h and 24 h post-byssal ablation, respectively. Meanwhile, proteomic analysis revealed 1254 and 484 differentially expressed proteins at the same two time points. Integrated analysis identified 121 genes differentially expressed at both transcript and protein levels. Among these genes, 44 were significantly upregulated, and they may constitute high-confidence gene sets associated with mussel byssogenesis. Notably, they included genes encoding tyrosinase-like protein, low affinity immunoglobulin epsilon Fc receptor, and O-methyltransferase MdmC. They were enriched in KEGG pathways, including metabolism of amino acids, lipid metabolism, nucleotide metabolism, and immune system. Quantitative real-time PCR was performed on seven selected genes, confirming that their expression patterns were consistent with those observed in transcriptomic and proteomic sequencing. This study provides novel data and insights for understanding the molecular basis involved in byssus development of *M. galloprovincialis*.

## 1. Introduction

*Mytilus galloprovincialis* Lamarck, 1819, a marine bivalve mollusk commonly found on rocky shores [1], is recognized for its high nutritional value, bioactive compounds, and substantial protein content [2]. Its remarkable adaptability has facilitated its establishment on a near-global scale, often outcompeting native *Mytilus* species in regions such as North America and Asia, which has made it a model organism for studying marine invasion dynamics [3,4]. Since its large-scale cultivation inception in the 1970s [5], it has become the fourth most cultivated shellfish species in China [6]. By 2023, the mussel farming area in China had expanded to 39,313 hectares, yielding 77,100 tons [7]. The rapid growth of mussel farming along China’s coast has led to overexploitation, resulting in suboptimal reproductive conditions, genetic degradation, germplasm erosion, and increased seedling detachment [5]. *Mytilus* species, including *M. galloprovincialis*, are globally important in mariculture [8], facing challenges like seasonal growth variations and biosecurity concerns, while byssal attachment strength is crucial for both aquaculture stability and understanding fitness and adaptation in wild populations [9].

Mussels adhere to diverse hard surfaces using byssus secreted by the byssal glands within the foot tissue [10]. Mussel foot tissue contains three glands: core (collagen) gland, cuticle (accessory) gland, and plaque (phenol) gland [11], which synthesize and store byssal precursor proteins [12]. The byssus comprises thread and adhesive plaques [13], primarily composed of mussel byssus proteins (MFPs) [14,15]. MFPs within the mussel byssal plaque rich in 3,4-dihydroxyphenylalanine (DOPA) can form cross-links with solid surfaces underwater, enabling attachment to various surfaces [16,17,18,19]. The key adhesive proteins include the thread matrix proteins (PTMP1, TMPs), the mussel foot proteins (Mfp-1, to -6), and the post-translational modification enzymes [20]. Mfp3 and Mfp5 are extensively researched proteins primarily situated at the base of the byssal plaque, where adhesion between mussel byssus and the substrate surface occurs. These proteins play a crucial role in adhesion within the byssus structure [21,22,23]. The mussel culture industry has experienced significant growth in recent years, leading to increased interest in mussel byssus within the aquaculture sector. Consequently, mussel byssus adhesion proteins have emerged as a focal point in biomaterial engineering research [24]. However, existing literature, both domestically and internationally, predominantly concentrates on the adhesion mechanisms and applications of byssus proteins, with limited exploration of the key genes and regulatory mechanisms governing the mussel byssus expression.

Despite this knowledge, the key genes regulating byssal protein expression and secretion within the foot tissue remain poorly understood. Transcriptomics and proteomics offer powerful tools for such investigations [25]. Successfully applied in related molluscan research. Sun et al. conducted a comprehensive analysis of specific genes and proteins in the fast and slow muscles of scallops [26]. Their study focused on distinguishing features between the fast and slow muscles of scallops, highlighting key components such as muscle contractible proteins, membrane and extracellular matrix, and enzymes of metabolic pathways. Similarly, Xu et al. delved into the interplay among calcium regulation, biomineralization, and yellow shell pigmentation in *Pinctada martensii* by employing transcriptomic and proteomic analyses to compare individuals with yellow and black shells [27]. Despite the widespread application of transcriptome and proteome sequencing techniques, there is a paucity of studies utilizing these methods to investigate the crucial genes involved in mussel byssus formation. This study employs transcriptome and proteome analysis of the foot tissue of *M. galloprovincialis* following byssal ablation to identify key genes and elucidate molecular regulatory mechanisms governing byssal secretion.

## 2. Results

### 2.1. Statistics of Transcriptome and Proteome Sequencing

Transcriptome sequencing was conducted on foot tissues of mussels at 0 h, 9 h, and 24 h post-cutting, yielding 627.36 million Clean Reads. Each sample exhibited a clean bases ranging from 65.74 to 84.37 million, with a Q30 base distribution of 94.78% to 95.64% and an average GC content of 43.46% (Table S1). Alignment rates to the reference genome ranged from 71.58% to 78.72%, (unique alignment rate ~63.97%). The findings affirm that the chosen reference genome satisfies the prerequisites for subsequent analyses.

The foot tissues of mussels were collected at 0 h, 9 h, and 24 h after byssus ablation for proteome sequencing. Following LC-MS/MS detection and database searching, comparing the signal intensity of the corresponding peptides in different samples, the proteins corresponding to the peptides were relatively quantified. Proteomics identified 9413 proteins across samples. A total of 110,223 peptides were quantified, providing robust proteome coverage.

### 2.2. Analysis of Differentially Expressed Genes (DEGs)

**Figure 1:**
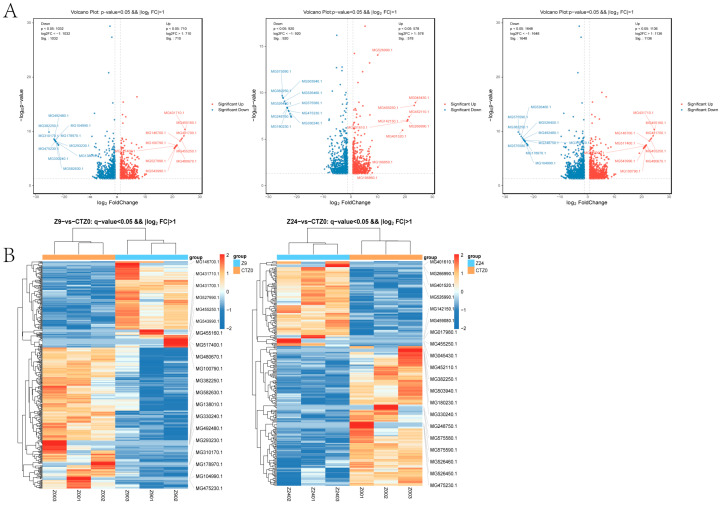
Differentially expressed genes among transcribed groups in foot tissue of mussel. (**A**). Volcano Plot of DEGs between 9 h vs. 0 h control, 24 h vs. 0 h control, and 9 h, 24 h vs. 0 h control. The dots in the graph represent transcripts that are significantly differentially expressed. Blue dots represent transcripts with significantly lower expression levels, while red circles represent transcripts with significantly higher expression levels (*p* < 0.05). &&: Both screening criteria were met in the figure (*p*-value < 0.05 and |log_2_FC| > 1.0). (**B**). Cluster heat map of differential gene expression among transcribed groups in foot tissue of mussel. &&: Both screening criteria were met in the figure (q-value < 0.05 and |log_2_FC| > 1.0).

A total of 1742 DEGs (Table S3) were identified in the foot transcription group at 9 h when compared with the 0 h control group (*p*-value < 0.05 and |log_2_FC| ≥ 1.0), of which 710 were up-regulated and 1032 were down-regulated (Table S3). There were 1498 DEGs at 24 h compared with the 0 h control group, including 578 up-regulated genes and 920 down-regulated genes. Volcano diagrams illustrate asymmetry in DEGs between up-regulated (red) and down-regulated (blue) genes (Figure 1A). The expression trends of DEGs at 9 h and 24 h are shown in Figure 1B. After removing duplicates, a total of 2789 DEGs were identified in the foot transcription group at either 9 h or 24 h, including 1138 up-regulated genes and 1651 down-regulated genes.

**Figure 2:**
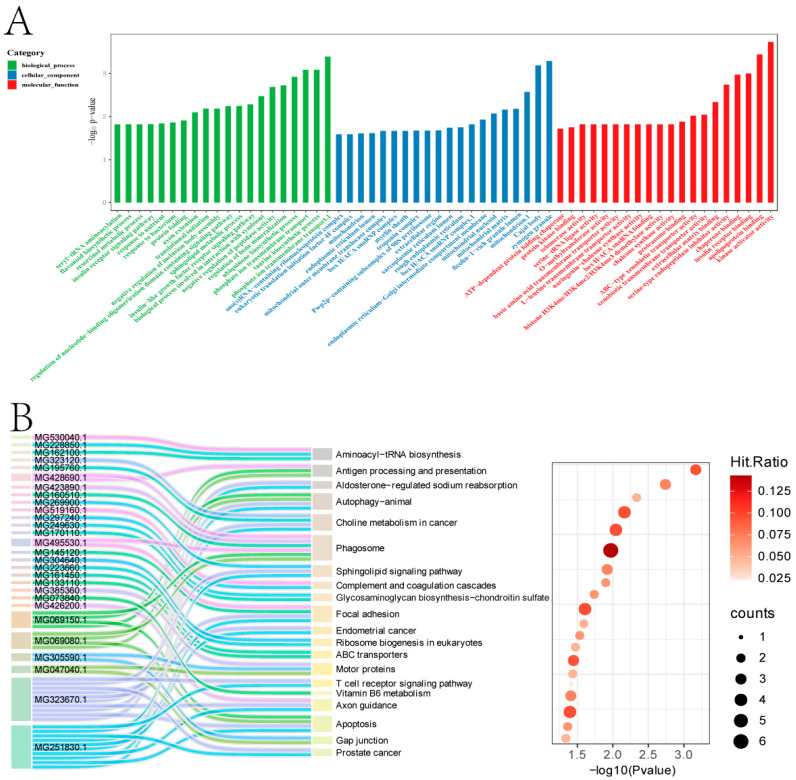
Functional analysis of the 1138 DEGs. (**A**). GO enrichment analysis. (**B**). KEGG enrichment analysis.

The 1138 up-regulated DEGs were annotated to 1333 GO subcategories (Figure 2A). GO enrichment analysis indicated that they were enriched in categories such as cytoplasm, nucleus, membrane, metal ion binding, extracellular space, plasma membrane, mitochondrion, ATP binding, extracellular region, mitochondrion, and identical protein binding, etc. KEGG analysis revealed that the 1138 up-regulated genes were distributed in 244 pathways (Figure 2B). The enriched pathways potentially associated with byssus secretion mainly included phagosome, autophagy—animal, focal adhesion, apoptosis, PI3K-Akt signaling pathway, antigen processing and presentation, axon guidance, and regulation of actin cytoskeleton, etc.

### 2.3. Analysis of Differentially Expressed Proteins (DEPs)

**Figure 3:**
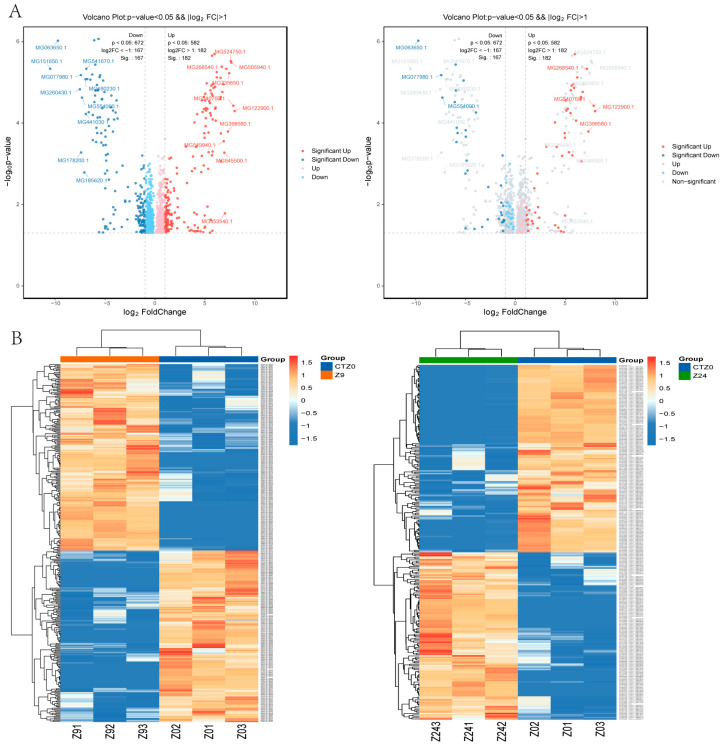
DEPs (differentially expressed proteins) in foot tissue of mussel. &&: Both screening criteria were met in the figure (*p*-value < 0.05 and |log_2_FC| > 1.0). (**A**). Volcano Plot of DEPs between 9 h vs. 0 h control, 24 h vs. 0 h control. The dots in the graph represent proteins that are significantly differentially expressed. Blue dots represent proteins with significantly lower expression levels, while red circles represent proteins with significantly higher expression levels (*p* < 0.05). (**B**). Cluster heat map of differential protein expression between groups of mussel foot tissue protein.

Quantitative proteomics based on iTRAQ identified 1254 differentially expressed proteins (DEPs) (*p*-value < 0.05, FC ≥ 2.0 and FC ≤ 1/2.0) in the 9 h group compared to the 0 h control group (582 up-regulated and 672 down-regulated DEPs); 484 DEPs were identified in the 24 h group compared to the 0 h control group (202 up-regulated and 282 down-regulated) (Figure 3A; Table S3). The expression trends of DEPs at 9 h and 24 h are shown in Figure 3B. After removing duplicates, a total of 1571 DEPs were detected in the foot proteome at either 9 h or 24 h, including 709 up-regulated proteins and 862 down-regulated proteins.

**Figure 4:**
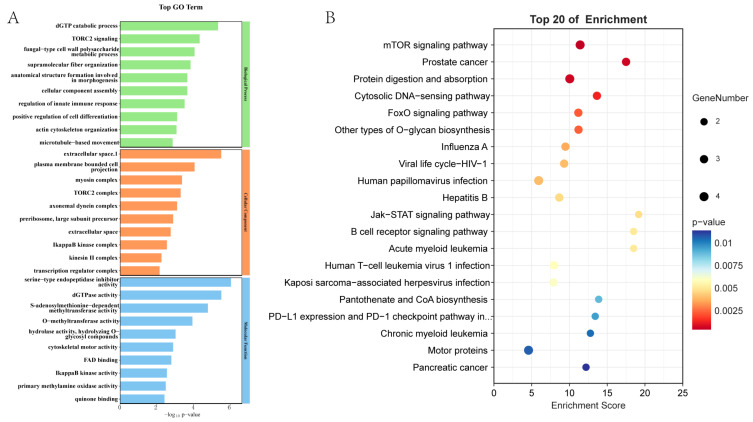
Functional analysis of the 709 DEPs. (**A**). GO enrichment analysis. (**B**). KEGG enrichment analysis.

The 709 up-regulated DEGs were annotated to 430 GO subcategories (Figure 4A). They were enriched in categories such as extracellular space, membrane, cytoplasm, plasma membrane, zinc ion binding, ATP binding, serine-type endopeptidase inhibitor activity, extracellular region, metal ion binding, GTP binding, and DNA binding, etc. KEGG analysis revealed that the 709 proteins were distributed in 243 pathways (Figure 4B). The enriched pathways included protein digestion and absorption, motor proteins, mTOR signaling, other types of O-glycan biosynthesis, cytosolic DNA sensing, cytoskeleton in muscle cells, etc.

### 2.4. Integrated Transcriptome-Proteome Analysis

**Figure 5:**
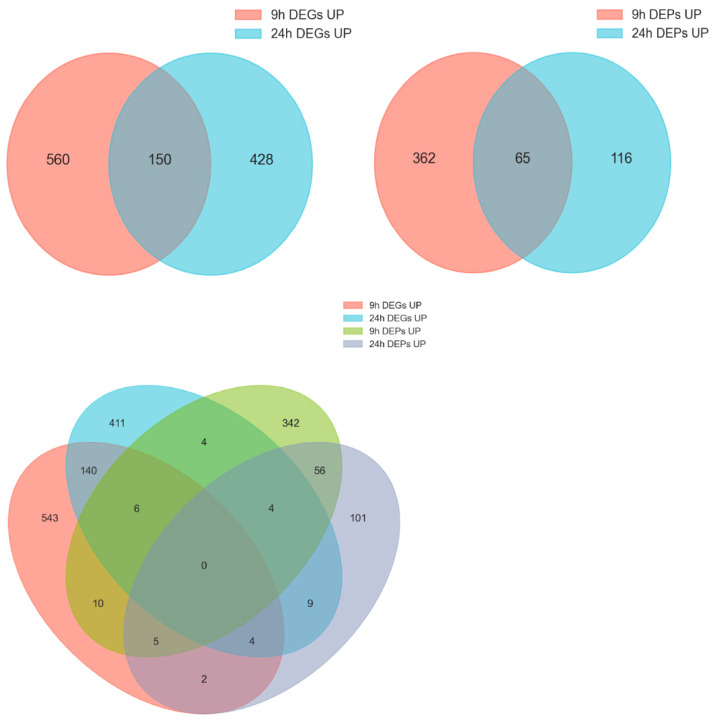
Joint analysis of the DEGs and DEPs in 9 h and 24 h samples.

Venn analysis identified 70 genes at 9 h and 51 genes at 24 h that were significantly differentially expressed at both the transcript and protein levels (Figure 5). Among these genes, 44 were up-regulated at both levels, and they may constitute high-confidence gene sets associated with mussel byssogenesis. The genes included putative epidermal cell surface receptor, low affinity immunoglobulin epsilon Fc receptor (FcεRII/CD23), cystine sulfinic acid decarboxylase, tyrosinase-like protein, O-methyltransferase MdmC, and post-GPI attachment to proteins factor 1, etc.

KEGG analysis of the 44 co-upregulated genes (Table S2) revealed that they were enriched in pathways including glycan biosynthesis/metabolism, metabolism of other amino acids, lipid metabolism, nucleotide metabolism, folding/sorting/degradation, immune system, etc.

### 2.5. qPCR Validation of 7 Genes with DEGs

**Figure 6:**
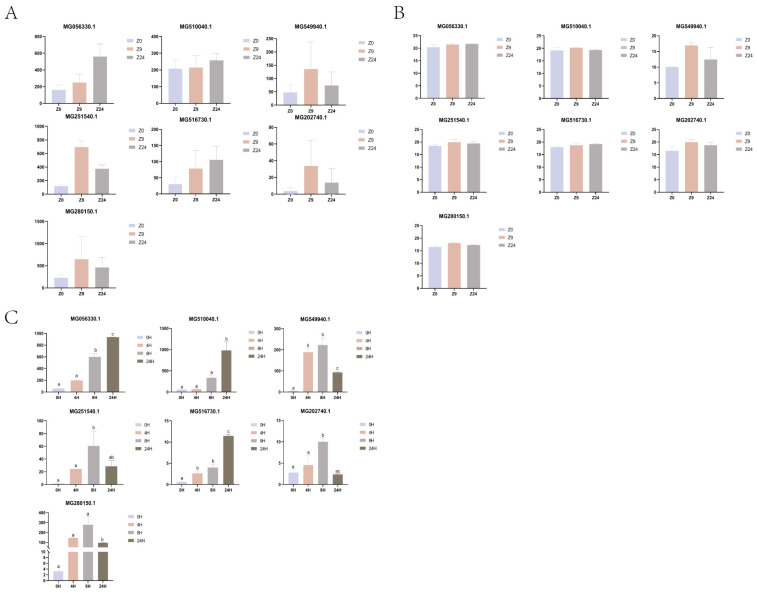
Expression patterns of seven selected genes. (**A**). The expression profiles based on transcriptomic data; (**B**). The expression profiles based on proteomic data; (**C**). qPCR analysis. a, b, c: Different letters indicate that the *p*-value between groups is <0.05.

The expression of 7 DEGs was verified by qRT-PCR (Figure 6). MG056330.1 was annotated as inter-alpha-trypsin inhibitor; MG510040.1 was annotated as reticulon-4 receptor; MG516730.1 was annotated as antithrombin-III. These three genes were up-regulated at both 9 h and 24 h after cutting the byssus. MG549940.1 and MG251540.1 were putative as low affinity immunoglobulin epsilon Fc receptor and tyrosinase-like protein, respectively. These two genes were up-regulated at both 9 h and 24 h compared with the 0 h control, but down-regulated at 24 h compared with the 9 h. MG202740.1 and MG280150.1 were not annotated, but their expression patterns were identical to the above two genes.

The qPCR expression patterns of all seven genes were consistent with those of transcriptome and protein sequencing.

## 3. Discussion

This study provides an integrated transcriptomic and proteomic analysis of *M. galloprovincialis* foot tissue during byssal regeneration, identifying key genes and pathways involved in this critical process.

The ECM-receptor interaction pathway is a signaling mechanism for extracellular matrix (ECM)-cell surface receptor engagement, supporting key cell biological functions like adhesion, migration, proliferation, and tissue repair [28]. In this study, the genes encoding four proteins in the pathway—cartilage oligomeric matrix protein (COMP, MG495530.1), podocan (PODN, MG495470.1), FRAS1-related extracellular matrix 2 (FREM2, MG395520.1), and collagen type VI alpha 3 (COL6A3, MG028220.1)—were significantly upregulated following byssus cutting, indicating their potential roles in byssogenesis. As a member of the thrombospondin protein family, COMP exerts a significant influence on the extracellular matrix (ECM) by regulating cell attachment [29], cell survival, and interactions with various ECM components [30]. Since byssus secretion depends on both ECM stability and cell attachment capacity [31], COMP may modulate byssus secretion by controlling ECM stability. PODN belongs to the small leucine-rich repeat protein family, contributing to extracellular matrix function and regulating cell–matrix interactions [32]. Additionally, PODN participates in cell signal transduction, which may influence cell proliferation and apoptosis [33]. When mussels secrete byssus, they regulate local pH and redox states to control the oxidation of byssus proteins and balance adhesion and crosslinking levels—this signal transduction mechanism may parallel PODN’s role in cell signaling. FREM2 is an extracellular matrix protein that facilitates cell adhesion to the matrix, participates in intracellular and extracellular signal transduction, influences cell growth, differentiation, and apoptosis [34], and may also affect the secretion and attachment of mussel foot proteins. The COL6A3 gene encodes the collagen type VI alpha 3 chain, critical for the stable assembly of type VI collagen. As a key component of the extracellular matrix, this alpha 3 chain provides physical support to cells and helps maintain tissue structural integrity [35]. Thus, COL6A3 may influence the secretion and adhesion of mussel foot proteins by regulating extracellular matrix composition and structure.

The Hippo signaling pathway, conserved across multiple species and implicated in environmental information processing, is crucial for regulating cell proliferation, apoptosis, and migration [36]. Within this pathway, two genes—encoding Merlin (MERL, MG434080.1) and Protocadherin Fat 4 (FAT4, MG308180.1)—were identified, and they may be associated with byssus secretion. The Merlin protein inhibits cell proliferation and promotes apoptosis by interacting with cytoskeletal proteins and cell membrane receptors [37]. We thus hypothesize that Merlin’s roles in cytoskeletal regulation and cell proliferation may indirectly influence byssus secretion. Protocadherin Fat 4 (FAT4), by contrast, potentially participates in diverse biological processes through regulating cell–cell adhesion and signal transduction. Although the precise function of the FAT4 gene has been primarily investigated at the cellular level [38], its involvement in cell adhesion and morphogenesis could also be indirectly linked to byssus development in mussels.

The inter-alpha-trypsin inhibitor (IαI, MG516730.1) is a complex containing the proteoglycan bikunin, whose family members have evolved over hundreds of millions of years during vertebrate evolution [39]. Members of the IαI family function in matrix tissues, cell signaling, protease inhibition, and regulation of complement activation, and are expressed in numerous tissues [40]. When bikunin is deficient, genes associated with stress, apoptosis, aging, cytokines, and hyaluronic acid metabolism become dysregulated [41]. IαI forms covalent complexes with hyaluronic acid via its heavy chains [42], which significantly enhances the mechanical strength and stability of the extracellular matrix. Notably, mussel foot proteins also rely on DOPA-mediated covalent crosslinking to solidify the byssus-matrix interface, sharing functional homology with IαI in the “ECM enhancement” mechanism.

The reticulon-4 receptor (RTN4R; MG510040.1) is a member of the nogo receptor family. By binding to inhibitory ligands, it can activate downstream signaling pathways (e.g., RhoA/ROCK), leading to growth cone collapse and blocked axonal regeneration [43]. Additionally, RTN4R plays a key role in nerve development and injury repair [44]; mutations in RTN4R may cause loss or enhancement of receptor function, thereby affecting nerve regeneration [45]. The upregulation of RTN4R in mussels following byssus cutting suggests that the injury repair process may be triggered, which is crucial for subsequent byssus development.

Antithrombin III (ATIII; MG516730.1) is a single-chain plasma glycoprotein belonging to the serine protease inhibitor (Serpin) superfamily. In vertebrates, it is one of the most critical natural anticoagulants, primarily functioning to inhibit key enzymes in the coagulation cascade—including thrombin and factor Xa—thereby regulating blood coagulation homeostasis and preventing pathological thrombosis [46,47]. While the canonical anticoagulant role of ATIII appears unrelated to mussel byssus development at first glance, its underlying molecular function as a serpin (i.e., protease inhibition) may provide a potential link. Byssus formation relies on the precise assembly of foot-secreted proteins, a process likely modulated by proteases that control protein cleavage, crosslinking, or degradation [48]. Thus, mussel ATIII homologs might regulate byssus matrix maturation by inhibiting specific proteases, making this gene a worthy target for further functional investigation.

The low-affinity immunoglobulin epsilon Fc receptor (FcεRII/CD23, MG549940.1) is a membrane-bound protein belonging to the immunoglobulin Fc receptor family. In vertebrate systems, it is best characterized for its role in type I hypersensitivity reactions (e.g., anaphylaxis) [49], functioning as a low-affinity receptor for immunoglobulin E (IgE). Specifically, FcεRII/CD23 regulates IgE production and B cell differentiation [50], and modulates adaptive immune responses by mediating IgE-dependent antigen uptake and presentation to T lymphocytes [51]. For mussels, byssus secretion is a key adaptive behavior enabling attachment to substrates, and this process may be linked to immune regulatory mechanisms [52]. Notably, when mussels experience byssus cutting—a stressor that disrupts their attachment—they may modulate immune responses to mitigate environmental stress (e.g., pathogen exposure at the site of byssus re-secretion or physiological stress from reattachment). In this context, mussel FcεRII/CD23 homologs could potentially participate in coordinating immune adaptation during byssus regeneration, though this functional link requires further investigation.

The tyrosinase-like protein (MG251540.1) is a copper-dependent metalloenzyme with wide distribution across microorganisms, animals, plants, and humans [53]. Canonical tyrosinases act as key enzymes in melanin biosynthesis, catalyzing the hydroxylation of L-tyrosine to L-3,4-dihydroxyphenylalanine (L-DOPA) [54]. For mussels, the catechol functional group of DOPA is recognized as a core component driving byssus adhesion: alkaline pH and cations in seawater oxidize these catechol groups, which then undergo further conversion into polymers. Stable substrate adhesion is ultimately achieved through chelation between these polymers and environmental metal ions [55]. Mussel foot proteins (MFPs) are rich in DOPA residues, and DOPA content is critical for the adhesive performance of the byssus. Specifically, studies have demonstrated a positive correlation: higher DOPA content in MFPs corresponds to stronger adhesive capacity of the byssus [56].

## 4. Materials and Methods

### 4.1. Animal Materials

Mussels (*M. galloprovincialis*) were collected from Rizhao, China (119.53° E, 35.42° N) in November 2024, with an average body weight of (11 ± 1.0) g and shell height of (46 ± 1.5) mm. Mussels were acclimated for 7 days in laboratory conditions, with daily feeding of Chlorella sp. and seawater replacement every 24 h. After completely severing the byssus of 40 randomly selected mussels along the shell edge using surgical scissors, the byssal secretion status was observed and recorded at 1, 2, 4, 6, 8, 12, and 24 h post-severing. Foot tissue was dissected at time points 0 h, 9 h, and 24 h post-severing, flash-frozen in liquid nitrogen, and stored at −80 °C.

### 4.2. Transcriptome Sequencing and Analysis

Total RNA was extracted using the TRIzol reagent (Invitrogen, Carlsbad, CA, USA) according to the manufacturer’s protocol. RNA purity and quantification were evaluated using the NanoDrop 2000 spectrophotometer (Thermo Scientific, Waltham, MA, USA). RNA integrity was assessed using the Agilent 2100 Bioanalyzer (Agilent Technologies, Santa Clara, CA, USA). At each time point, equal amounts of foot RNA from 3 mussels were pooled to form one biological replicate, with 3 biological replicates established. Then the libraries were constructed using the VAHTS Universal V6 RNA-seq Library Prep Kit (NR616-02, Vazyme, Nanjing, China) according to the manufacturer’s instructions. Transcriptome sequencing was conducted by OE Biotech Co., Ltd. (Shanghai, China). Libraries were sequenced on an Illumina Novaseq 6000 platform (San Diego, CA, USA), generating 150 bp paired-end reads.

Raw sequencing reads were processed using fastp (Version: 0.20.1) [57] for quality control, and low quality reads were removed to obtain clean reads. The clean reads were mapped to the reference genome using HISAT2 (Version: 2.1.0) [58]. Read counts of each gene were obtained by HTSeq-count (Version: 0.11.2) [59], and transcript expression levels for each gene were quantified as FPKM values [60]. Differential expression analysis was performed using DESeq2 (Version: 1.22.2) [60]. Genes with a *p* value < 0.05 and log_2_|FoldChange| > 1 were defined as significantly differentially expressed genes (DEGs). The comparison of mean expression levels at two time points for both the transcriptome and proteome was conducted using a t-test, which was performed using the R programming language. Hierarchical cluster analysis of DEGs was performed to demonstrate the expression pattern of genes in different groups and samples. GO [61] and KEGG [62] enrichment analyses of DEGs were performed to screen for significantly enriched terms and pathways, respectively.

### 4.3. Proteome Sequencing and Analysis

Tissues identical to those used for transcriptome sequencing were adopted for proteome sequencing. Briefly, tissues were homogenized in a mixture containing a final concentration of 1 mM of protease inhibitor (PMSF, Biyuntian, ST507-10 mL) and phosphatase inhibitor (Phosphatase Inhibitor Cocktail A (50X), Biyuntian, P1082). The protein concentration in each sample was determined using a BCA protein assay kit (ThermoScientific, 23225). 10 μg of protein from each sample were separated by 12% SDS-PAGE, stained and rinsed for 15 min, and finally scanned using an automatic digital gel image analysis system (Tianneng1600, Tanon, Shanghai, China) [63]. Subsequently, the DIA-NN software (Version: 1.8.1) was employed to integrate all quality-filtered data for DIA quality data database search and protein DIA quantitative analysis [64]. The search was performed against the Uniprot proteome database for *Mytilus galloprovincialis* (UP000596742, downloaded 25 March 2025). The key parameters for DIA-NN analysis were configured as follows: trypsin was specified as the digestion enzyme with a maximum of one missed cleavage permitted. Carbamidomethylation of cysteine (C) was set as a fixed modification, while oxidation of methionine (M) and acetylation of protein N-terminus were designated as variable modifications.

The database search was conducted using a target-reverse strategy. Both peptide-spectrum matching (PSM) false discovery rate (FDR) and protein FDR were set at 0.01 (1%), ensuring high confidence in protein identification and quantification. GO [61] and KEGG [62] enrichment analyses of DEPs were performed to screen for significantly enriched terms and pathways, respectively. These experimental procedures were performed by OE Biotech Co., Ltd. (Shanghai, China).

### 4.4. Joint Analysis Transcriptome and Proteome Data

Integrated analysis was conducted using genes derived from transcriptomic and proteomic data. Specifically, the intersection of differentially expressed genes identified at the transcript and protein levels was selected and designated as a high-confidence gene set associated with byssogenesis in mussels.

### 4.5. Quantitative Real-Time PCR (qPCR) Validation

To validate the gene expression patterns observed in our omics analyses, qPCR was employed to assess the relative mRNA expression levels of genes that were significantly upregulated in mussel foot tissue following byssus ablation. Seven genes were selected for RT-qPCR analysis, with three biological replicates performed for each gene. Primers for qPCR were designed using Primer Premier 6 software (Table 1), with EF1α [65] serving as the internal reference gene. All primers were synthesized by Qingdao Ruibo Xingke Biotechnology Co., Ltd. (Qingdao, China). The relative expression levels of genes across groups were determined using the 2^−ΔΔCt^ method. For the intergroup comparison of q-PCR data, we used ANOVA for statistical testing, which was performed using GraphPad Prism 9.5 software.

## 5. Conclusions

In this study, transcriptomic and proteomic analyses elucidated the complex molecular response underlying byssal regeneration in *M. galloprovincialis*. Key genes associated with byssogenesis included those involved in extracellular matrix stabilization (e.g., IαI-like), DOPA synthesis (e.g., tyrosinase-like), cellular signaling (e.g., RTN4R-like and FcεRII-like protein-encoding genes), and protease regulation (e.g., antithrombin-III-like). Additionally, genes involved in essential metabolic pathways—known to support the energy and substrate demands of byssal formation—were also identified as integral to this process. These findings provide a crucial molecular framework for understanding byssus development, with implications for improving mussel aquaculture practices through targeted manipulation of adhesion and potentially inspiring novel bioadhesive designs.

## Figures and Tables

**Table 1:** Primers used for qRT-PCR.

Gene	Primer	Sequences (5′–3′)	Amplicon Length
EF1α	F	CGTTTTGCTGTCCGAGACATG	92 bp
	R	CCACGCCTCACATCATTTCTTG	
MG056330.1	F	TGAGACAGATAGCAATGGTT	172 bp
	R	ACAGTAGATAGGACAAGATGG	
MG251540.1	F	TTCTCTGCGTCTCATTATCA	191 bp
	R	GTTATTGCCTCTGTCATAGC	
MG510040	F	GATTGTGTTGGACCTAGTATG	140 bp
	R	TGCCATTGTCTTCTGCTAT	
MG549940.1	F	TCTTCGTTGATTCACATCCA	167 bp
	R	TTCCACGCAACTTCCATAT	
MG516730.1	F	GATAGACGATGCCACTGT	145 bp
	R	TATGCTCATCATGTCAACTG	
MG202740.1	F	TTGACAATGAAGAACGGAAC	109 bp
	R	GCACACCAGCATCTATACA	
MG280150.1	F	TGGATATGGTGGTTACAGAA	186 bp
	R	GTTAAGGTACTACTTCGTATGG	

## Data Availability

Data will be made available on request.

## Supplementary Materials

The following supporting information can be downloaded at: https://www.mdpi.com/article/10.3390/ijms262110511/s1.
